# Big Five Personality Traits, Coping Strategies and Compulsive Buying in Spanish University Students

**DOI:** 10.3390/ijerph18020821

**Published:** 2021-01-19

**Authors:** José Manuel Otero-López, María José Santiago, María Cristina Castro

**Affiliations:** Department of Clinical Psychology and Psychobiology, Faculty of Psychology, C/Xosé María Suárez Núñez, s/n, Campus Vida, 15782 Santiago de Compostela, Spain; mariajose.santiago@usc.es (M.J.S.); mariacristina.castro@usc.es (M.C.C.)

**Keywords:** big five personality traits, coping strategies, compulsive buying, university students

## Abstract

Personality traits and coping strategies have historically been two key elements in the field of health psychology. It is, therefore, striking that there is no study in the field of compulsive buying that integrates the most generic, decontextualized and stable aspects (traits) with those having a more marked processual and dynamic nature, which are closer to goal-based views of human nature (coping strategies). Another weakness of the compulsive buying field is that, despite the confirmed growing increase in compulsive buying in the younger age groups, most studies have been conducted with adult samples. Hence, this study seeks to clarify the role of the Big Five domains and different coping strategies in university students’ compulsive buying. The sample consisted of 1093 participants who were classified as either compulsive buyers or non-compulsive buyers. Both groups were compared regarding sociodemographic variables (gender, age), the Big Five personality traits, and coping strategies through chi-square tests or Student’s *t*-tests. Besides, a multivariate logistic regression analysis was conducted to determine which of these determinants might play a part in the construction of a risk profile for compulsive buying. The results showed that other than gender (specifically being female), Neuroticism and the use of such coping strategies as problem avoidance and wishful thinking are risk factors that increase the propensity for compulsive buying. The use of active coping strategies such as problem solving, cognitive restructuring and social support, as well as the Conscientiousness dimension are protection factors that decrease the likelihood of becoming a compulsive buyer. Finally, and on the basis of the findings obtained, possible guidelines are given, which, hopefully, may effectively contribute to the prevention of and/or intervention in compulsive buying among young adults.

## 1. Introduction

There is a broad consensus in the scientific literature on the fact that compulsive buying has become, over the last few decades, a growing phenomenon that is characteristic of modern consumer societies. It has been conceptualized as a chronic and repetitive purchasing that becomes a primary response to negative events or feelings that provides short-term positive rewards but results in long-term negative consequence [1] both personally and within the family [2,3]. The age of onset of compulsive buying tends to occur by the late teen years or early twenties [4].

In a recent meta-analysis [5] the estimated rate of prevalence of compulsive buying was 4.9% in the representative adult general populations, whilst for university student samples, prevalence rates were higher (8.3%). Another very recent study [6] obtained very high percentages of prevalence of compulsive buying in samples from university students from several countries (21.3% USA, 16% China, 16.1% South Korea). Studies conducted in Spain have obtained percentages of prevalence of compulsive buying of 7.1% in the general population [7], and 7.4% in university students [8]. These data seem to place university students’ compulsive buying as a research focus, thus making the design of prevention and/or intervention programs with assured efficacy a challenge for the foreseeable future. The identification of the possible causes that elicit, maintain, or promote the purchasing behavior at this age bracket seems to be way forward.

In this sense, it is a known fact that the university stage entails ecological transition [9] in the life of young adults (new educational challenges, new interpersonal relations, greater economic independence, greater decision-making capacity, less supervision of spending behavior, greater degrees of conscientiousness) which, most likely, will sometimes become sources of stress [10,11,12] and may be accompanied by some negative emotionality [8,13]. The personality traits and how these young individuals cope with things that happen to them become extremely relevant at this age bracket. It is precisely within this context that this study is framed. It seeks to integrate two types of explanatory units which, in the light of some proposals [14,15], would occupy different “spaces” or “levels”.

On the one hand, there are the traits labelled by McCrae and Costa [14] as “basic tendencies” (within a framework that also includes “characteristic adaptations”) and placed by McAdams [15] at Level I within a three-level proposal (Levels II and III being Personal concerns and Life stories, respectively). In both schemes, traits are viewed as broad dimensions of personality, endogenous, comparative, stable and hierarchically structured.

Research suggests that personality traits may play a critical role in the vulnerability of some persons to develop compulsive buying [16]. The Five Factor Model [17], the most widespread perspective on the study of the human trait structure, postulates five basic personality domains (Neuroticism, Extraversion, Openness, Agreeableness and Conscientiousness). It has had an important heuristic value both in general and clinical samples of population in the field of compulsive buying. There are differential patterns of association with compulsive buying on the basis of the trait being analyzed. The findings of previous research, regardless of the type of sample used, are highly consistent as regard to the fact that while Neuroticism (tendency to experience negative emotions such as anxiety and depression) is a risk factor, Conscientiousness (tendency to show self-discipline and to act dutifully) acts as a protective factor against compulsive buying [18,19,20]. As to the relationship between Agreeableness (tendency to altruism, trust, modesty, and cooperativeness) and compulsive buying, findings are disparate as some studies report a positive relation [19,21] whilst other studies report a negative relation [22,23,24]. As to the Openness (tendency towards curiosity, creativity and preference for novelty and variety) and Extraversion (tendency to be sociable, warm, active, assertive, cheerful, and in search of stimulation) traits, findings are still rather unclear and, in most studies, associations are weak [20,25]. Although traits are relatively stable across culture [26,27], discrepancies in findings may be explained, at least partially, by the fact that the studies that link the Big Five to compulsive buying in university students have been made in different countries, such as the USA [19], Norway [22], Taiwan [28], Pakistan [25], and Malaysia [24]. In this regard, in a study conducted across 56 nations [29], in which most samples consisted of college students, cross-cultural differences in the Big Five (particularly in Openness and Conscientiousness) were confirmed. In this context, and as there are no studies that relate the Big Five and compulsive buying in Mediterranean Countries of Southern Europe such as Spain, conducting this study with university students will provide new data on this issue. Be that as it may, throwing light on the role that traits play in compulsive buying is as yet an unfinished task and consequently one that needs further research.

On the other hand, and apart from the traits that some authors have labelled as the “having” side of personality [30], this study includes coping in order to allow for a more dynamic, active, intentional construct that may represent the manner in which traits materialize in a specific environment to which the “characteristic adaptations” [14,31] refer to coping, which has been described as “personality in action under stress” [32] (p. 525), would also have a place in the so-called “middle-level units” [33] in reference to what personality “does”, to the intentions and efforts that govern people’s behavior, and contextualized in time, situation and social roles.

Specifically, coping conceptualized as “cognitive and behavioral efforts to manage specific external and/or internal demands that are appraised as taxing or exceeding the resources of the person” [34] (p. 141), has attained remarkable prominence in the areas of physical and mental health [35]. It is however surprising in this regard that as far as the field of compulsive buying is concerned, it has received little attention. This circumstance is all the more remarkable if we consider that in other areas related to impulse control and/or behavioral addictions such as pathological gambling [36,37,38], eating disorders [39,40], internet addiction [41,42] or Facebook addiction [43,44], coping has been widely explored. Besides, and given that compulsive buying has been characterized as a maladaptive mean of alleviating negative emotions [45], it is surprising that only two studies [7,46] have explored in depth what specific strategies the individuals with this problem use. The findings in these studies suggest that passive coping strategies of problem avoidance, wishful thinking, and self-criticism constitute risk factors for compulsive buying [7], and that the tendency to compulsive buying is associated with maladaptive mental disengagement, denial and lack of acceptance coping strategies [46].

In this context, delving into which the “specific” coping strategies are that characterize university students who are vulnerable to compulsive buying is yet another focus of our study, an objective that is in line with the transactional perspective of stress in Lazarus and Folkman [34] which emphasizes that individuals are not characterized by the use of a specific type of strategy, but instead they resort to a wide array of strategies.

In sum, to our knowledge, there is no study that has brought together the Big Five dimensions of personality and different types of coping strategies to better understand compulsive buying in university students. Specifically, this study has the following objectives: (a) to elucidate whether the Big Five personality dimensions significantly discriminate between compulsive buyers and non-compulsive buyers, (b) clarify what type of coping strategies differentiate at statistically significant levels between compulsive buyers and non-compulsive buyers and (c) establish a risk profile for compulsive buying in university students which considers both personality traits and coping strategies.

## 2. Materials and Methods

### 2.1. Procedure

This article falls within the framework of a wider research project conducted during the last decade with the purpose of exploring the nature and the extent of compulsive buying as well as its connection with personal and social variables in the region of Galicia (Spain). The underlying idea, given the difficulty of following up participants over time, was to select every four years a new large cross-sectional cohort and evaluate the prevalence and the impact of a number of variables in different samples. In another words, while in the early studies we used representative samples of the general population, in more recent years, and since little is known in this field of work about the phenomenon in young samples, we have evaluated samples of university students. Specifically, and following a cross-sectional design, the sample in this study was collected during the 2017–2018 academic year (between November and March), among students from different schools of the University of Santiago de Compostela (Spain). Prior to the handing out of questionnaires, we contacted some professors from different schools who gave us the opportunity of presenting our research to their students and those who voluntarily accepted to do so filled out the battery of self-reports during the class. Participants received paper-versions of questionnaires, and precise information on how to complete them. Inclusion criteria for this research were as follows: being a student over 18 years of age, a fluent Spanish speaker, not currently under psychopharmacological treatment or psychotherapy, and having no current impulse control disorder other than compulsive buying. In addition, a written consent form was obtained, and the confidentiality of the data was guaranteed. The study was conducted in accordance with the Declaration of Helsinki, and the protocol was approved by the Bioethics Committee of the University of Santiago de Compostela. The percentage of participation was 97.8%.

### 2.2. Participants

The sample included in this research was comprised of 1093 university students. As for gender, there were 571 females (52.2%) and 522 males (47.8%). The age ranged from 18 to 23 years (M_age_ = 19.49, SD = 1.31). As for field of study, 29.1% of participants were Science students, 30.6% Health students, and 40.3% Social Sciences and Law students. The respondent’s field of study did not result in any statistically significant differences (*Χ*^2^ = 1.12, *p* = 0.569) in compulsive buying. Table 1 depicts data corresponding to the frequencies and percentages of the characteristics according to gender and age, for the whole sample and for the groups of compulsive buyers and non-compulsive buyers.

### 2.3. Measurements

#### 2.3.1. Compulsive Buying

The tendency to compulsive buying was measured by the German Addictive Buying Scale (GABS) [47], in its Spanish translated version (GCBS) [48]. This questionnaire has 16 items (e.g., “I often feel a sudden, inexplicable urge to go out immediately and buy things that I want”, “I often buy things just because they’re cheap”, “I’ve often bought things that I’ve later not used”) which should be answered on a scale from 1 (strongly disagree) to 4 (strongly agree). The total score (range: 16–64) is considered as an indicator of compulsive buying tendency with higher ratings representing a higher tendency to compulsive buying and vice versa. GCBS has previously demonstrated adequate psychometric properties in other research carried out with Spanish samples [49,50]. In this study, Cronbach’s alpha value was 0.89. In keeping with the objectives of this study, and in accordance with some previous studies [51,52], we adopted a cut-off score that was two standard deviations above the mean value of the group in GCBS. Given that the mean GCBS score in the total sample was 31.21, and the standard deviation was 7.13, a mark of 45 was taken as the cut-off score for classifying subjects as compulsive buyers. This cut-off score matches those in previous studies [7,8,53].

#### 2.3.2. Big Five Personality Traits

The Big Five dimensions were assessed using the Spanish version [54] of the Big Five Inventory (BFI) [55]. This self-report includes 44 short phrases measuring the core features of the Big Five. Items are introduced with the stem “I see myself as someone who…” and the endings change in order to assess the distinct personality traits: Neuroticism (e.g., “Gets nervous easy”), Extraversion (e.g., “Is outgoing, sociable”), Openness (e.g., “Is ingenious, a deep thinker”), Agreeableness (e.g., “Is helpful and unselfish with others”) and Conscientiousness (e.g., “Makes plans and follows through with them”). Statements are answered on a 5-point Likert scale (1 = disagree strongly, 5 = agree strongly). BFI has been used in Spain in previous studies in this field of work [50]. In this study, Cronbach’s alphas for the Big Five personality traits ranged from 0.79 for Conscientiousness to 0.85 for Neuroticism.

#### 2.3.3. Coping Strategies

The Spanish version [56] of the Coping Strategies Inventory (CSI) [57] was employed to evaluate eight coping strategies. Four of them were engagement coping: problem solving (e.g., “I changed something so that things would turn out alright”), cognitive restructuring (e.g., “I tried to get a new angle on the situation”), express emotions (e.g., “I let out my feelings to reduce the stress”), social support (e.g., “I accepted sympathy and understanding from someone”); and four were disengagement coping: problem avoidance (e.g., “I slept more than usual”), wishful thinking (e.g., “I wished that the situation had never started”), self-criticism (e.g., “I was my mistake and I needed to suffer the consequences”), social withdrawal (e.g., “I kept my thoughts and feelings to myself”). This measure consists of 40 items—five for each coping strategy—that should be answered on a scale of frequency ranging from 1 (never used) to 5 (always used). Adequate psychometric properties have been previously obtained with American [58] and Spanish samples [59]. In the present study, the coefficients of internal consistency (Cronbach’s alpha) for the coping strategies ranged between 0.79 (wishful thinking) and 0.93 (cognitive restructuring).

### 2.4. Statistical Analyses

Statistical analyses were conducted using IBM-SPSS Statistics software, version 24. (IBM, Armonk, NY, USA). In accordance with our main objectives, university students were classified into two groups: compulsive buyers and non-compulsive buyers. These two groups were compared for age, gender, the Big Five personality traits, and coping strategies using chi-square test for the categorical variable gender, and Student’s *t*-test for continuous variables. In determining which of these determinants constituted significant predictors of compulsive buying, the variables that were significantly related to this phenomenon at level *p* ≤ 0.05 in the univariate analyses were then included in a multivariate logistic regression analysis (Enter method). As to the collinearity diagnosis among these selected variables, it should be noted that the tolerance level was 0.55–0.89 (greater than 0.1), whilst the range of the Variance Inflation Factor (VIF) was 1.11–1.80 which was less than 10, indicating absence of multicollinearity [60]. The odds ratio (OR) and the 95% confidence intervals (CI) of the ORs were calculated. In addition, the Wald statistic was used to determine significance of predictors and the Nagelkerke’s R^2^ to give account of the percentage of explained variance in compulsive buying.

## 3. Results

Comparisons between compulsive buyers and non-compulsive buyers in relation to sociodemographic determinants (see Table 1) revealed significant prevalence differences by gender, as compulsive buying prevalence was higher in females than males (10.5% and 5%, *Χ*^2^ = 11.49, *p* = 0.001). As to age, comparison results showed that the two groups did not differ at statistically significant levels.

Another objective of this study was to explore which domains of the Five-Factor Model established significant differences between compulsive and non-compulsive buyers. The results of *t*-test (Table 2) confirmed that these groups differ significantly in Neuroticism (*t*(1091) = −7.49, df = 1091, *p <* 0.001), Conscientiousness (*t*(1091) = 7.00, *p* < 0.001) and Agreeableness (*t*(1091) = 2.47, df = 1091 *p <* 0.014). Specifically, compulsive buyers obtained significantly higher scores than the comparison group with respect to Neuroticism, and lower scores in Conscientiousness and Agreeableness. As far as the remaining traits, it should be noted that compulsive buyers score higher than non-compulsive buyers in Extraversion and lower in Openness, but differences between both groups do not reach statistical significance in either case.

Findings obtained from the comparison between the groups in relation to coping (Table 2) revealed that all the coping strategies considered (except express emotions and social withdrawal) established statistically significant differences. Specifically, compulsive buying scored significantly higher on the passive coping strategies of problem avoidance, wishful thinking, and self-criticism (*t* values ranging from −12.39 to −7.67, *p* < 0.001), and significantly lower (*t* values ranging from 3.67 to 7.32, *p* < 0.001) on the active-focused on the problem coping strategies of problem solving, cognitive restructuring, and social support.

Lastly, in order to establish a risk profile for compulsive buying, a logistic regression analysis was conducted considering the compulsive buying status (0 = non-compulsive buying, 1 = compulsive buying) as the criterion variable, and the variables which in the univariate analyses of variance made it possible to differentiate between compulsive buyers and non-compulsive buyers at significant levels (namely, gender, Neuroticism, Agreeableness, Conscientiousness, problem solving, cognitive restructuring, social support, problem avoidance, wishful thinking, and self-criticism) as predictors. As it can be seen in Table 3, all variables considered, with the exception of Agreeableness and self-criticism, were significant predictors of compulsive buying. Specifically, it should be noted that a large amount of compulsive buying variance (Nagelkerke’s R^2^ = 0.457) was explained for by the variables in the model.

Our results confirm that being female, Neuroticism, and the passive-avoidance coping strategies of problem avoidance and wishful thinking acted as a risk factor for compulsive buying. By contrast, the determinants that were found to act as protective factors against this phenomenon was one dimension of the Big five (Conscientiousness), and the active coping strategies of problem solving, cognitive restructuring, and social support.

## 4. Discussion

Personality traits and coping have become in the last few years two extremely interesting topics for social sciences and health researchers. In the specific field of compulsive buying, although there are indeed studies analyzing personality traits, very few studies analyze coping and, so far, no study has brought together both determinants to advance our understanding of this behavioral problem. Thus, the core objective of this study is to elucidate which Big Five Dimensions and which coping strategies constitute risks and protective factors against compulsive buying in university students.

The comparison between compulsive buyers and non-compulsive buyers in the Big Five domains of personality (first objective of this study) shows that the compulsive buyers group scores, at statistically significant levels, higher in Neuroticism and lower in Conscientiousness and Agreeableness. 

As to Neuroticism, these results are in line with some previous studies conducted both in general population samples [18,20,21], and in clinical populations [61] which link emotional instability to compulsive buying. The lower score in Conscientiousness of the compulsive buyers’ group is also consistent with that obtained in other studies, which not only indicates lower levels of Conscientiousness in compulsive buyers [19,20,28], but also in individuals that are vulnerable to impulse buying [62,63]. Additional evidence of this pattern of results also comes from recent studies that highlight the role of Neuroticism and Conscientiousness in other addictions such as food addiction [64], Facebook addiction [65], Internet addiction [22], addictive smartphone use [66] and problem gambling [67]. Lastly, as far as Agreeableness is concerned, and despite the fact that previous findings with regard to this dimension are more inconsistent, our results are in line with those obtained in some previous research [18,20,24] that suggest that compulsive buyers are less agreeable. Be that as it may, the fact is that studies conducted in neighboring fields such as Internet addiction [68] or smartphone addiction [69] report a negative covariance between Agreeableness and these addictions, a finding that, albeit indirectly, is consistent with our findings.

The exploration of differences in coping strategies between students who are compulsive buyers and those who are not compulsive buyers (second objective) constitutes one of the main strengths of this study. It is noteworthy that according to our results compulsive buyers use a markedly maladaptive style. They report overuse of strategies involving “not doing” (passive-avoidance coping strategies) and a prominent misuse of the active coping strategies (which involve doing something to find a solution). Specifically, our findings confirm, on the one hand, that compulsive buyers tend, to a greater extent than non-compulsive buyers, to use disengagement strategies, which are not aimed at taking action or change, and which reflect a lack of ability or a rejection to perceive situations differently such as avoidance, wishful thinking and self-criticism. On the other hand, the erratic management of coping strategies is exacerbated if we take into account that, as we mentioned above, compulsive buyers, when compared with non-compulsive buyers, face stressful events using to a lesser extent markedly proactive strategies such as problem solving, cognitive restructuring and seeking social support.

In general, these results are in line with those for other behavioral addictions such as pathological gambling [37,70] or Internet addiction [71], that showed that in behavioral addictions there are maladaptive coping styles.

The search for a pattern of risk and protective factors in university students of Galicia with compulsive buying behavior by taking jointly both the traits (Big Five domains) and different coping strategies is, as we have noted, the core objective of this study. The results show that, as well as gender (namely, being female), Neuroticism and the use of such coping strategies as problem avoidance and wishful thinking are risk factors in relation to compulsive buying. The use of active coping strategies such as problem solving, cognitive restructuring and social support, as well as Conscientiousness, are protective factors against this behavioral addiction.

In order to further our understanding of these findings, we should begin by highlighting that gender emerges as an important determinant of compulsive buying, as suggested by a great deal of studies, which point to the higher vulnerability of women for extensive shopping patterns [2,72,73]. As to traits, two dimensions of the Five-Factor Model play a significant role in the vulnerability profile of university students to compulsive buying: Neuroticism and Conscientiousness.

As far as Neuroticism is concerned, it could be hypothesized that the high degree of vulnerability to stress and the marked sensitivity to negative outcomes that characterizes this trait could act, at least partially, as a “trigger” for compulsive buying intention, this behavior thus becoming a “reliever of emotional distress” and a way of coping-with-stress. Elaborating on this line of argument, if only tentatively, a possible route of influence could be negative emotionality—stress—compulsive buying. Be that as it may, this idea is not completely novel in the field as under Baumeister’s escape theory [74,75] buying behavior can be understood as an attempt to escape negative feelings by focusing attention on external stimuli such as buying; the early and widely accepted definition of compulsive buying by O’Guinn and Faber [1] also alluded to the fact that individuals engage in compulsive buying as a means of alleviating negative feelings. Whatever the route of influence may be, the fact is that many studies have characterized compulsive buyers as emotionally unstable, experiencing negative emotions such as anxiety or depression to a greater extent than non-compulsive buyers [6,51,76], as well as having higher levels of impulsivity [77,78].

Low Conscientiousness constitutes, as shown by the results, a risk factor for compulsive buying. This finding has already been widely reported in the previous literature [19,28]. A recent study [20] that analyzed the different facets of this trait noted that individuals with a high propensity for compulsive buying showed low scores on the dutifulness and self-discipline facets. It is revealing, in this regard, the thesis by Faber and Vosh [79] who hold that “compulsive buying represents a form of self-regulatory failure” (p. 547). Additional evidence on the importance of this trait or its facets in a variety of aspects (money control, debt, behavioral addictions) comes from a number of studies. It has been confirmed that Conscientiousness affects efficient money management [80]. Self-control mitigates the link between compulsive buying and debt [72] and was the most significant predictor not only of compulsive buying but also of problematic Internet and mobile phone use in college students [81].

Coping strategies emerge, as well as traits (Neuroticism and Conscientiousness) prominently in the configuration of the profile of a university student with compulsive buying behavior. Our findings confirm that problem avoidance and wishful thinking increase the likelihood of participants being classified as compulsive buyers (risk factors). Elaborating on these results, it should be noted that dysfunction in avoidance behavior lies, at least partially, in that whilst it may help restore “calmness” in the short term, it consolidates a very powerful reinforcer of the behavior to be avoided; what seems to be the solution to distress becomes the problem. Wishful thinking (e.g., “I had fantasies or wishes about how things might turn out”, “I wished that the situation had never started”) indicates a vigilant and obsessive-ruminative style of processing information. On the contrary, the use of strategies that try to lead to change (problem solving), that change the meaning of stressful transactions taking into account their positive aspects (cognitive restructuring), or that they facilitate the emotional support of the significant others (social support) are, according to our results, protective factors against the development of the compulsive buying in university students.

Unfortunately, the scarcity of previous studies analyzing coping and compulsive buying prevents a more in-depth discussion of these findings. In any case, our results are in line with the two studies we are aware of that note that, on the one hand, mental and behavioral disengagement are linked to this problem [46], and that, on the other hand, individuals showing a higher propensity to compulsive buying resort more frequently to passive coping strategies [7]. Supplementary evidence relating to impulse buying is provided by the study by Yi and Baumgartner [82] who confirm that the shame experienced following impulse buying episodes is associated with the use of avoidant coping strategies.

Finding that there is an overuse of disengagement coping strategies by compulsive buyers would, among other things, allow for the thesis that advocates that compulsive buying is a form of escape from negative affect states [83], or for those holding that the acquisition of objects may compensate for, reward or neutralize negative feelings [84]. The mood-repairing function of compulsive buying, that is postulated by the “*self-medication*” hypothesis [85], or even the fact of considering “*a strong materialistic value orientation as one way in which people attempt to compensate for worries and doubts about their self-worth, their ability to cope effectively with challenges, and their safety in a relatively unpredictable world*” ([86], p. 14) also fall under this line of argument.

Be that as it may, our results place the Neuroticism-coping strategies pairing as a fundamental piece to complete the puzzle of the dynamics underlying compulsive buying. Most likely, scoring high in Neuroticism increases the probability of using maladaptive strategies (e.g., problem avoidance, wishful thinking) and reduces the probability of using adaptative regulation strategies (problem solving, cognitive restructuring and social support). Some facets of Neuroticism (e.g., anxiety, depression, vulnerability to stress) will probably have an impact not only on a more negative assessment of the stressing events but also on the ability to face them appropriately. It may be also postulated that the low Conscientiousness of compulsive buyers implies a lower probability of using strategies leading to actively face the problem; the literature reports a negative association between Conscientiousness and the denial coping strategies [87,88]. Our findings also support the notion that the profile consisting of high Neuroticism, low Conscientiousness and disengagement coping is one particularly toxic in the compulsive buying behavior of students. Given the lack of studies in the field of compulsive buying, and in order to support this later consideration, it seems appropriate to cite the studies conducted in adolescents with Internet addiction [89,90] which confirm the influence of the Neuroticism and Conscientiousness traits and the emotion-centered coping strategies in explaining Internet addiction.

### 4.1. Strengths and Limitations

This study has major strengths which we believe contribute to a better understanding of compulsive buying. Choosing university students as the focus of research is a deliberate decision for this research that goes beyond the large sample size it provides. The university stage often entails many changes and challenges that cause significant levels of stress in young individuals. This circumstance is sometimes accompanied by the emergence of certain psychosocial problems such as eating disorders, alcohol and/or drug abuse, which means that studying the way young individuals face things that happen to them seems necessary to understand and explain their behavior. The analysis of the personal characteristics or traits that make a specific individual more vulnerable to engaging in compulsive behavior is yet another way of approaching the analysis. Indeed, in this study, including a wide array of coping strategies (that constitute an example of units-of-action that involve “doing something”) jointly with the Big Five (basic dispositions of personality) to progress in the knowledge of what the protective and risk factors are, from these domains is an important gain. This knowledge will also make it possible to postulate new suggestions for (preventive and/or intervention) action to stop and/or inhibit the emergence of compulsive buying at this evolutionary stage.

Of course, this research has its limitations. Firstly, this was a cross-sectional design so causal effects cannot be established. Therefore, longitudinal studies should be conducted in order to capture the dynamics and influences (which will be probably reciprocal) among the constructs analyzed. Secondly, although the self-reports have been widely used in the literature to evaluate compulsive buying and a number of associated variables, evaluation could be improved with data collection from multi-method and multi-informant methods. Thirdly, the inequality of sample size between non-compulsive and compulsive. Fourthly, using a sample of Spanish students means that we should be very cautious when generalizing the results to other types of samples and socio-cultural environments. Lastly, the constructs considered in this study are just some of the many that could have been analyzed in the study of vulnerability to compulsive buying in university student samples. Thus, the experience of stress situations in different domains (family relational, academic), the support perceived and/or received from a variety of social agents (parents, friends, professors) or the feeling of solitude could be just some examples.

### 4.2. Further Research

Future research faces some important challenges in elucidating the nature and scope of the dynamics taking place between the traits of individuals with buying disorders and their strategies to cope up with environmental demands they find stressful. This study has merely attempted to build a starting point in this direction. However, there are plenty of aspects that may be included in an agenda for further research. In the light of the results regarding the Big Five, and specifically, the role of Neuroticism and Conscientiousness, elucidating which facets of each dimension contribute the most to the prediction of compulsive buying at this age bracket will provide a finer and more complete analysis. Also highly beneficial for the field would be to conduct further studies in this age bracket to define the baseline from which action may be taken, adding other personality variables beyond traits (units of action such as personal projects, strivings, life tasks, for example) so that we are able to gain a more complete picture of the influences. Including more specific coping strategies, evaluating the role of the nature-frequency-severity of the stressor, clarifying the role of gender, age and evolutionary stage, are also other suggestions for the future. A final and perhaps more important demand is the need to conduct longitudinal studies that are able to capture not only stability and change, but also shed light on the role (antecedent, mediator…) of the different variables involved in compulsive buying in order to better clarify this behavioral problem. The Big Five (or their facets) as exogenous determinants and the coping strategies with a mediating role channeling the influence of traits in compulsive buying, could be, in view of the available evidence, a good starting point to advance in the empirical verification of the causal approaches so that we can not only better understand the dynamics among variables of different levels, but also promote the design of action programs with greater assurances of success.

## 5. Conclusions and Implications

The present study, from the integration of the basic dimensions of personality (specifically, the Big Five personality traits) and coping (different coping strategies), extends our knowledge of the risk and protective factors of compulsive buying among university students. Results clearly show that both types of determinants are necessary and useful to attain a better understanding of compulsive buying. Thus, comparisons between compulsive buyers and non-compulsive buyers confirm the existence of statistically significant differences in almost all the variables examined. Specifically, differences were found for gender, a variety of personal dimensions (Neuroticism, Conscientiousness and Agreeableness), different active strategies (problem solving, cognitive restructuring and social support) and passive coping strategies (problem avoidance, wishful thinking and self-criticism).

The results show that, as well as the gender (namely, being a woman), Neuroticism and the use of such coping strategies as problem avoidance and wishful thinking, constitute risk factors in relation to compulsive buying. The use of active coping strategies such as problem solving, cognitive restructuring and social support, as well as Conscientiousness, constitute protective factors against this behavioral addiction.

Finally, the present study has practical implications, as the findings obtained reveal the urgent need to look not only at personality traits (Neuroticism and Conscientiousness), but also at the misregulation of coping strategies which characterizes compulsive buyers. Thus, in the light of our results, striving to promote the well-being of young individuals, reducing their negative emotions and educating in responsibility are just, on the basis of personal dimensions, some guidelines and suggestions for action. More specifically, our results encourage the design and implementation of prevention and intervention programs for compulsive buying, which act at different levels and make it possible that young individuals may (a) efficiently face and manage negative emotions (e.g., anxiety, depression, hostility) associated to the purchasing behavior; (b) acquire specific skills to identify stress and be able to successfully handle highly stressful situations; (c) encourage certain personal facets (deliberation, discipline, sense of competence) which increase their control over their behavior (e.g., to be able to resist the strong urge and need to buy something to feel better), and (d) analyze their goals (academic, interpersonal, family…) and acquire skills to plan their behavior and, in short, promote their wellbeing and self-efficacy. Besides, the centrality of the use of passive coping strategies as a risk factor for compulsive buying necessarily entails making young individuals aware of the need to play an active role in the management of their problems. Awareness that some specific strategies such as avoidance or denial rather than a solution to the problem often end up being “the problem” may be a good start. Teaching and training in proactive coping styles (e.g., problem solving, cognitive restructuring) will encourage a planned behavior and self-control which, most likely, will contribute, albeit indirectly, to a reduction of young individuals’ negative emotionality. These suggestions, among many other possible ones, merely seek to be a wake-up call for the need to “do something” on the face of a growing psychosocial problem in modern consumption societies.

## Figures and Tables

**Table 1:** Comparison among Non-CB and CB as for sociodemographic characteristics.

	Total Sample *n* = 1093		Non-CB *n* = 1007		CB *n* = 86		Comparison Non CB vs. CB
	*n*	%	*n*	%	*n*	%	
**Gender**							*Χ*^2^ = 11.49, *p* = 0.001
Male	522	47.8	496	49.3	26	30.2	
Female	571	52.2	511	50.7	60	69.8	
**Age**	Mean	SD	Mean	SD	Mean	SD	*t* = −0.448, *p* = 0.654
	19.49	1.31	19.48	1.32	19.55	1.21	

Note. Non-CB = Non-Compulsive Buyers; CB = Compulsive Buyers.

**Table 2:** Comparison among Non-CB and CB as for traits and coping strategies.

	Total Sample *n* = 1093		Non-CB *n* = 1007		CB *n* = 86		T	*p*
	Mean	SD	Mean	SD	Mean	SD		
*Traits*								
Neuroticism	26.13	5.47	25.77	5.36	30.27	5.10	−7.496	<0.001
Extraversion	26.53	5.51	26.51	5.51	26.81	5.49	−0.493	0.622
Openness	36.05	5.65	36.09	5.69	35.59	5.57	0.780	0.436
Agreeableness	31.14	4.30	31.24	4.27	30.05	4.53	2.472	0.014
Conscientiousness	29.28	5.61	29.62	5.50	25.30	5.49	7.001	<0.001
*Coping strategies*								
Problem solving	14.87	3.34	15.08	3.19	12.40	4.06	7.329	<0.001
Cognitive restructuring	15.59	3.56	15.72	3.42	14.12	4.65	4.046	<0.001
Express emotions	13.85	3.80	13.89	3.64	13.41	5.38	1.124	0.261
Social support	17.10	4.81	17.25	4.69	15.28	5.79	3.674	<0.001
Problem avoidance	13.27	3.42	12.92	3.13	17.38	4.02	−12.395	<0.001
Wishful thinking	14.74	3.77	14.39	3.54	18.88	4.01	−11.187	<0.001
Self-criticism	13.50	3.80	13.25	3.59	16.44	4.80	−7.678	<0.001
Social withdrawal	12.03	3.34	12.00	3.34	12.41	3.33	−0.077	0.282

Note. Non-CB = Non-Compulsive Buyers; CB = Compulsive Buyers.

**Table 3:** Results of the logistic regression analysis with compulsive buying as dependent variable.

	B	S.E.	Wald	*p*	OR	95% CI
Gender (male = 0, female = 1)	−0.846	0.305	7.690	0.006	0.429	0.236–0.780
Neuroticism	0.148	0.030	24.757	0.000	1.160	1.094–1.230
Agreeableness	−0.007	0.032	0.041	0.839	0.993	0.928–1.058
Conscientiousness	−0.060	0.027	4.959	0.026	0.942	0.893–0.993
Problem solving	−0.164	0.053	9.518	0.002	0.849	0.765–0.942
Cognitive restructuring	−0.099	0.047	4.480	0.034	1.104	1.007–1.210
Social support	−0.097	0.028	11.969	0.001	0.908	0.860–0.959
Problem avoidance	0.239	0.045	28.059	0.000	1.270	1.162–1.387
Wishful thinking	0.171	0.050	11.494	0.001	1.187	1.075–1.310
Self-criticism	0.041	0.038	1.157	0.282	1.042	0.967–1.123

Note. Nagelkerke’s R^2^ = 0.457.

## Data Availability

The data present in this study are available on request from the corresponding author. The data are not publicly available due to privacy restrictions.
